# Extended Spectrum β-Lactamase-Producing *Escherichia coli* from Poultry and Wild Birds (Sparrow) in Djelfa (Algeria), with Frequent Detection of CTX-M-14 in Sparrow

**DOI:** 10.3390/antibiotics11121814

**Published:** 2022-12-14

**Authors:** Mohamed Belmahdi, Nadia Safia Chenouf, Abdelkrim Ait Belkacem, Sandra Martinez-Alvarez, Mario Sergio Pino-Hurtado, Zahra Benkhechiba, Samiha Lahrech, Ahcène Hakem, Carmen Torres

**Affiliations:** 1 Department of Biology, Faculty of Natural and Life Sciences, University of Djelfa, Moudjebara Street, Djelfa 17000, Algeria; 2 Biochemistry and Molecular Biology, Faculty of Science and Technology, University of La Rioja, 26006 Logroño, Spain; 3 Laboratory for Exploitation and Development of Steppe Ecosystems, FNLS, University of Djelfa, Moudjebara Street, Djelfa 17000, Algeria; 4 Department of Agronomy, Faculty of Natural and Life Sciences, University of Djelfa, Moudjebara Street, Djelfa 17000, Algeria; 5 Center for Research in Agropastoralism, University of Djelfa, Moudjebara Street, Djelfa 17000, Algeria

**Keywords:** *Escherichia coli*, ESBL, turkey, sparrow, CTX-M-14, Algeria

## Abstract

Antimicrobial resistance is a global threat that is spreading more and more in both human and animal niches. This study investigates the antimicrobial resistance and virulence threats of *Escherichia coli* isolates recovered from intestinal and fecal samples of 100 chickens, 60 turkeys, and 30 sparrows. Extended spectrum β-lactamase (ESBL) producing *E. coli* isolates were recovered in 12 of the animals tested, selecting one isolate per positive animal: sparrow (eight isolates, 26.7%), turkey (three isolates, 5%), and chicken (one isolate, 1%). The *E. coli* isolates were ascribed to B1 and D phylogenetic groups. The *bla*_CTX-M-14_ gene was detected in all ESBL-producing *E. coli* isolates from sparrow. The *bla*_CTX-M-15_ (two isolates) and *bla*_CTX-M-14_ genes (one isolate) were detected in the isolates of turkey, and the *bla*_CTX-M-1_ gene in one isolate from broiler. Three lineages were revealed among the tested isolates (ST/phylogenetic group/type of ESBL/origin): ST117/D/CTX-M-1/broiler, ST4492 (CC405)/D/CTX-M-15/turkey, and ST602/B1/CTX-M-14/sparrow. All isolates were negative for *stx1, sxt2*, and *eae* virulence genes. Our findings provide evidence that the sparrow could be a vector in the dissemination of ESBL-producing *E. coli* isolates to other environments. This study also reports, to our knowledge, the first detection of *bla*_CTX-M-14_ from sparrow at a global level and in turkey in Algeria.

## 1. Introduction

The value of antibiotics in human and veterinary medicine is of great importance in reducing morbidity and mortality of infectious diseases, in addition to improving animal production. Unfortunately, the effectiveness of these molecules does not last forever following the emergence of bacterial strains resistant to antibiotics due to the abuse of the latter [1]. The development of antibiotic resistance is of global relevance. Indeed, after their initial selection and their local diffusion, resistant bacteria can be transferred across international borders by humans (travelers), animals and insects (vectors), agricultural products, and water [2]. The appearance and spread of extended spectrum β- lactamase (ESBL) producing *E. coli* isolates is increasingly reported in both human and veterinary medicine. The phenomenon of resistance is not limited to domestic or farm animals but has also been detected in wild animals and birds, being birds in some cases used as indicators of the spread of resistance in the environment [3,4]. Several studies have been carried out on farm animals, such as broilers, laying hens, pigs and fish, or pets, whether in Algeria or worldwide [5,6,7,8]. Currently, a special interest has arisen in the study of antibiotic resistance in wild animals [3,9]. In the clinical niche, the ESBLs most frequently found in *E. coli* are of the CTX-M type, particularly CTX-M-15, CTX-M-14, and CTX-M-3. As a matter of fact, these types of ESBLs are the most frequently detected in Algeria either causing infections or colonizing patients [10,11]. On the other hand, other types have been detected in the other species of enterobacteria [12]. In the last years, the most prevalent and representative CTX-M-1 and CTX-M-9 group enzymes are CTX-M-15 and CTX-M-14, respectively [13]. The CTX-M-14 was first detected in a Chinese hospital in 1997 [14]. Since then, it has been increasingly reported. Currently, these two variants are known to be widely disseminated in human clinical samples [15], animals [16], and food-producing animals [17].

Wild birds play an important role in the dissemination of resistance to antibiotics either as carriers of resistant bacteria or of resistance genes. Moreover, they can act as propagators of these resistances due to their ability to migrate over long distances for short periods of time [18,19].

For this reason, we were interested in the study of antimicrobial resistant *E. coli* isolates recovered from sparrow, which is a migrating bird native of Algeria. The house sparrow is traditionally associated with human habitation. In urban areas, it is the most widely answered bird. In central and northern Europe, the population of this and of other urban bird species has declined. There are many theories as to why this decline occurred, but the lack of data on the number of house sparrows before their decline has hampered efforts to study these theories in detail. Among these theories we have parasitic infections, and changes in habitat which present, as an ecological perspective some novel challenges for birds, such as new predators, new microbiota, human presence, unique food resources, and high levels of chemical, light, and acoustic pollution [20]. The detection of *Campylobacter* in sparrows that live close to industrial farms opens the possibility of sparrow being a potential source of contamination of the chicken with these microorganisms. Nevertheless, the existence of quinolone resistant isolates in sparrow suggests their origin in the industrial farms (like chicken, pig, and cows) [21]. However, Dolejska et al. (2008) indicated that although house sparrows lived together with the cattle and came into contact with cattle waste on the farm, they were not infected by resistant *E. coli* isolates with the same characteristics as those found in cattle [22]. It is reasonable to believe that wild birds can also carry antibiotic resistant bacteria long enough during migration with the potential of intercontinental spread of resistance [19]. Chuma et al. (2000) show the possibility of transference of resistance between farm animals and the sparrow that live in the close areas. These genes can be transferred from *E. coli* to other bacterial species such as *Campylobacter*, which is considered one of the most pathogenic bacteria for humans [21]. In Algeria, three types of sparrow populations are observed. In addition to the two species most widely known as house sparrows (*Passer domesticus*) and the Spanish sparrow (*P. hispaniolensis*), a third hybrid form of the two previous species named as the Italian sparrow (long known phenotypically in North Africa by hybrid sparrows), is found in the Italian peninsula and some Mediterranean islands [23]. 

This study analyses the antibiotic resistance in *E. coli* isolates from Spanish sparrow in parallel with *E. coli* isolates obtained from broilers and turkeys (farm animals). To our knowledge this is the first study concerning the detection of ESBL-producing *E. coli* in this type of migratory bird which is of special relevance in Algeria.

## 2. Material and Methods

### 2.1. Sampling and Strain Isolation

A total of 190 intestinal and fecal samples were collected from 100 broilers (recovered from 4 poultry houses), 60 turkeys (recovered from a slaughterhouse and a turkey farm), and 30 sparrows, in Djelfa and M’sila cities (Algeria), between 2017 and 2019. Fecal samples were inoculated in nutrient broth (BHIB) at 37 °C for 24 h for enrichment. After that, enriched culture was seeded on Hektoen agar plates (Pasteur Institute of Algeria) supplemented with 2 µg/mL of cefotaxime and incubated for 24 h at 37 °C to recover cefotaxime-resistant (CTX^R^) *E. coli* isolates. Isolates with typical *E. coli* morphology were selected (one isolate per sample), identified by classic biochemical tests, and confirmed using the matrix-assisted laser desorption and ionization time-of-flight mass spectrometry (MALDI-TOF MS) with Biotyper software for bacterial identification (Bruker Daltonics, Bremen, Germany).

### 2.2. Antimicrobial Susceptibility Testing

Antimicrobial susceptibility was performed on Mueller–Hinton agar by standard disk diffusion procedure as described by the Antibiogram Committee of the French Society for Microbiology [24]. In this assay, a standard 0.5 McFarland inoculum was prepared and swabbed onto the surface of Muller-Hinton plates. Filter papers impregnated with a standardized concentration of antibiotic agent were placed on the agar surface. Antibiotics tested were as follows: amoxicillin–clavulanic acid (AMC), cefoxitin (FOX), cefotaxime (CTX), ceftazidime (CAZ), aztreonam (ATM), cefepime (FEP), imipenem (IMP), tobramycin (TOB), gentamicin (GEN), nalidixic acid (NAL), ciprofloxacin (CIP), tetracycline (TET), and trimethoprim-sulfametoxazole (STX) (Bioanalyse). In the same conditions, *E. coli* ATCC 25922 was used as a control strain. After incubation at 37 °C for 24 h, the diameter of the zone of inhibition around the disc was measured. The results were then interpreted according to the AC-FSM breakpoints [24]. *E. coli* isolates were finally classified as resistant (R), susceptible (S), or intermediate (I) for each of the antimicrobials tested. 

### 2.3. Phenotypic ESBL Detection

A screening test for extended spectrum β-lactamases (ESBL) production was carried out on Mueller–Hinton agar using the double disc synergy test (DDST) by placing disks of CAZ (30 µg) and CTX (30 µg) at a distance of 20 mm center to center from an amoxicillin-clavulanic acid disk (30 µg). In the same conditions, *K. pneumoniae* ATCC 700603 (an ESBL producer) was adopted as a positive control strain. An extension of the edge of the inhibition zone of the third generation cephalosporins disks (CAZ and/or CTX) in proximity to the AMC disk indicates a positive ESBL production [25].

### 2.4. DNA Extraction

For each *E. coli* isolate grown overnight on BHI agar plates, one colony was suspended in 1 mL of sterile milli-Q water. The cells were lysed by heating at 100 °C for 10 min, and cellular debris was removed by centrifugation at 12,000× *g* for 2 min. The supernatant was used as the source of template for PCRs [5].

### 2.5. Molecular Detection of Antibiotic Resistance Encoding Genes

β-lactamases-encoding genes *bla*_TEM_ (F-ATTCTTGAAGACGAAAGGGC; R-ACGCTCAGTGGAACGAAAAC; amplicon size 1150 bp), *bla*_SHV_ (F-CACTCAAGGATGTATTGTG; R-TTAGCGTTGCCAGTGCTCG; amplicon size 885 bp), *bla*_CTX-M-1_ group (F-GTTACAATGTGTGAGAAGCAG; R-CCGTTTCCGCTATTACAAAC; amplicon size 1041 bp), and *bla*_CTX-M-9_ group (F-GTGACAAAGAGAGTGCAACGG; R-ATGATTCTCGCCGCTGAAGCC; amplicon size 857 bp) were tested by PCR in ESBL-producing isolates and amplicons obtained were sequenced using the Sanger method [26,27]. Nucleotide segments and their deduced amino acid sequences were compared with those included in the GenBank database as well as with those deposited at the web site http://www.lahey.org/Studies/ (accessed on 31 October 2019), in order to ascribe the specific type of β-lactamase encoding gene. Positive and negative controls were included in all PCR assays. The presence of *mcr-1* gene (F-AGTCCGTTTGTTCTTGTGGC; R-AGATCCTTGGTCTCGGCTTG; amplicon size 320 pb) associated to colistin resistance, was tested by PCR in all ESBL-producing isolates. Moreover, the genes encoding resistance to tetracycline *tetA* (F-GTAATTCTGAGCACTGTCGC; R-CTGCCTGGACAACATTGCTT; amplicon size 937 pb) and *tetB* (F-CTCAGTATTCCAAGCCTTTG; R-CTAAGCACTTGTCTCCTGTT; amplicon size 416 pb), and those encoding resistance to sulfonamides *sul1* (F-TGGTGACGGTGTTCGGCATTC; R-GCGAGGGTTTCCGAGAAGGTG; amplicon size 789 pb) and *sul2* (F-CGGCATCGTCAACATAACC; R-GTGTGCGGATGAAGTCAG; amplicon size 722 pb) were also investigated by PCR [26,27]. *E. coli* isolates carrying the tested genes, detected by our team in previous studies [3,5,26,27], and PCR mix without bacterial DNA were used, respectively, as positive and negative controls in all cases.

### 2.6. Detection of Virulence Genes

The presence of the *eae* gene encoding the intimin, responsible for the attaching and effacing lesions of enteropathogenic *E. coli* isolates (EPEC) was tested by PCR (F-TCAATGCAGTTCCGTTATCAGTT; R-GTAAAGTCCGTTACCCCAACCTG; amplicon size 482 pb) [28]. Moreover, the shiga-toxin encoding genes *stx1* (F-CAGTTAATGTGGTGGCGAAGG; R-CACCAGACAATGTAACCGCTG; amplicon size 348 pb) and *stx2* (F-ATCCTATTCCCGGGAGTTTACG; R-GCGTCATCGTATACACAGGAGC; amplicon size 584 pb) were tested for the detection of shiga-toxin-producing *E. coli* isolates (STEC), as outlined by [29].

### 2.7. Detection and Characterization of Integrons

The presence of the *intl1* gene encoding the integrase of class 1 integrons was examined by PCR in all ESBL-producing isolates [26,27]. 

### 2.8. Phylogenetic Grouping and Multilocus Sequence Typing (MLST)

The ESBL-producing isolates were assigned to phylogenetic groups A, B1, B2, or D using a PCR strategy with specific primers for *chuA, yjaA*, and *TspE4.C2* genes, as previously described [30]. The MLST was performed on one *E. coli* isolate of each ESBL-type by amplification and sequencing of the seven housekeeping genes (*adk, fumC, gyrB, icd, mdh, purA* and *recA*) according to the *E. coli* MLST database (http://mlst.warwick.ac.uk/mlst/dbs/Ecoli, accessed on 31 November 2019).

## 3. Results

### 3.1. Bacterial Isolation

Among the fecal and intestinal samples of the 190 sparrows, broilers, and turkeys analyzed, 27 of them contained CTX^R^ *E. coli* isolates (five from broiler, 12 from turkey, and 10 from sparrow), and 12 animals harbored ESBL-producing *E. coli* isolates (6.3% of total samples). The prevalence ESBL-*E. coli* per animals tested was as follows: sparrow (eight out of 30, 26.7%); turkey (three out of 60, 5%) and chicken (one out of 100, 1%). One ESBL-producing *E. coli* isolate per positive sample was selected for further studies, making a collection of 12 isolates (Table 1).

### 3.2. Antimicrobial Resistance Phenotype

The results of antibiotic susceptibility of the 12 ESBL-producing *E. coli* isolates revealed that most of the isolates showed resistance to ciprofloxacin and tetracycline (>90%), 33.3% were resistant to tobramycin, and 16.7% to trimethoprim-sulfamethoxazole. All isolates showed susceptibility to cefoxitin, imipenem, and gentamicin.

### 3.3. Antimicrobial Resistance Gene Detection and Virulece Gene Content

All the eight ESBL-producing *E. coli* isolates originating from sparrows contained the *bla*_CTX-M-14_ gene. Likewise, the *bla*_CTX-M-14_ gene was also detected in one ESBL-producing *E. coli* isolate from turkey. The remaining two ESBL-producing *E. coli* isolates from turkey contained the *bla*_CTX-M-15_ gene. The *bla*_CTX-M-1_ gene was present in the ESBL-producing isolate of chicken. Resistance to tetracycline was mainly mediated by the *tetA* gene, except in one CTX-M-15-producing isolate from turkey in which the *tetB* gene was detected. Moreover, the integrase of class 1 integrons (*intl1*) was present in 10 out of the 12 ESBL-positive isolates.

All 12 ESBL-producing isolates were negative for the *stx1*, *stx2*, and *eae* virulence genes.

### 3.4. Multilocus Sequence Typing (MLST) Results

ESBL-positive isolates were ascribed to two phylogenetic groups: B1 (n = 8, all of them from sparrow) and D (n = 4, all of them from turkey and chicken). Regarding MLST typing, which was performed for three representative selected isolates (on the basis of the type of the ESBL-type and the origin of isolates), three lineages were revealed (ST/phylogenetic group/type of ESBL/origin): ST117/D/CTX-M-1/chicken, ST4492 (CC405)/D/CTX-M-15/turkey, and ST602/B1/CTX-M-14/sparrow.

## 4. Discussion 

*Escherichia coli* is a commensal microorganism of the digestive tract of humans and animals that may easily develop resistance under the selective pressure of antibiotics. It is also considered an opportunistic pathogen for humans and animals as well as a potential vector of resistance genes that could be transmitted to other pathogenic bacteria. It is qualified for these reasons as “indicator bacteria” [31]. Antibiotic-resistant *E. coli* isolates and resistance genes can be transferred to humans by the food chain or through direct contact with humans or animals, among other transmission routes [32]. Worldwide, the farming ecosystem is open and an exchange of resistant bacteria between different ecological niches is occurring at local, regional, national, and international levels due to farming and export systems. Moreover, resistant strains can be transported inside and outside the farm by people, birds, rodents, insects, water, and food [33].

Antibiotic-resistant *E. coli* isolates have been described in many species of wild birds. The detection of ESBL-producing *E. coli* isolates from wild birds was first reported in 2006 in Portugal [34]. Thereafter, many other reports have continued in Europe and around the world [19]. Among the CTX-M enzymes, CTX-M-15 and CTX-M-14 are the most frequently reported, in the human, animal and environment niches over the world [35]. This led us to focus our research on the genetic characterization of ESBL-producing *E. coli* isolates obtained from wild birds (sparrow) as well as poultry (chicken and turkey). Our results revealed that the frequency of ESBL-producing isolates among sparrow fecal samples (26.7%) was higher than those from chicken or turkey fecal samples (1% and 5%, respectively). All ESBL-producing *E. coli* isolates from sparrow carried the gene encoding CTX-M-14 and belonged to phylogenetic group B1. Similarly, the same ESBL type was detected in one isolate from turkey (*bla*_CTX-M-14_/B1), while the two remaining isolates contained the *bla*_CTX-M-15_ gene (phylogroup D). This study represents the first report of *bla*_CTX-M-15_ from turkey in Algeria. This ESBL variant has been reported among clinical *E. coli* isolates in many hospitals worldwide including Algeria [36,37]. The *bla*_CTX-M-1_ gene variant was also detected in the chicken isolate. 

The epidemiology of ESBL has changed worldwide in recent years. In the clinical environment, the most frequent ESBLs found in *E. coli* are the CTX-M type, particularly CTX-M-15 and in some countries CTX-M-3. On the other hand, other types have been detected in other enterobacteria species [12]. In chicken, the ESBL-producing *E. coli* with CTX-M-15 and CTX-M-1 variants were detected for the first time in 2012 from lesions of avian colibacillosis (broiler) in the region of central Algeria [38]. It was also detected in fish [39], in healthy cats and dogs [8] as well as in wild boars and barbarian macaques [9]. Recently, the CTX-M-15 was detected from food liver samples [27]. Around the world, CTX-M-15 has been detected in other types of birds such as the Gull-lipped Gull (France, Czech Republic and Sweden), Blackbird, White-fronted Goose, White-breasted Pigeon (Germany), Duck mallard, Herring gull (Poland), Gray-winged gull CTX-M-15 and CTX-M-14 has been also detected (Russia), pig (Japan) [40], and broiler (France) [41,42,43,44,45,46].

It is of interest the detection of the *bla*_CTX-M-14_ gene in fecal *E. coli* isolates from turkey and in sparrow. To our knowledge, this is the first time that CTX-M-14 is described in sparrow and turkey in Algeria. This type of ESBL was previously revealed in turkey in Great Britain [47], and in other types of animals, such as birds of prey, wild birds (*Larus* sp.), Black-headed warbler (Portugal), Black-headed gull (Sweden), broiler chicken (Spain, Japan and Tunisia), from cattle and bovine (England) [48], and from healthy or diseased food-producing animals (pig, duck, and goose among others) [17,34,41,44,49,50]. The CTX-M-14 was first detected in a Chinese hospital in 1997 [17]. In Algeria, the first report of CTX-M-14 was from *Salmonella enterica* serotype Kedougou in clinical isolates at Bejaia city [51] then, from *S. enterica* in Tizi Ouzou hospital [52], as well as among *E. coli* isolates from Tlemcen Hospital [53].

Three different sequence types were detected among our ESBL-producing isolates: ST4492/CC405 (*bla*_CTX-M-14_, turkey), ST602 (*bla*_CTX-M-14_, sparrow), and ST117 (*bla*_CTX-M-1,_ broiler). The lineages ST117 and ST405 were detected in *bla*_CTX-M-15_-producing *E. coli* isolates in many Algerian environments: in hospitals [53], in wild boars [9] and agricultural land [54]. Likewise, they were reported from domestic ducks in Bangladesh [4]. In Tunisia, ST405 was associated with CTX-M-8 and ST602 associated with CTX-M-1 in *E. coli* isolates recovered from food [48]. Moreover, the *E. coli* ST602 was isolated from the Andean condor [55] and from dogs [56]. The lineage ST117 is known to be frequently detected in broiler [57].

Antibiotics have been used in animals for therapy, prophylaxis, and also during many years as growth promoters (GP). Nevertheless, the use of antibiotics as GP has been banned in many countries of the world, although it is still allowed in many others (26% of the 160 world countries analyzed), according to the OIE [58]. The use of antibiotics for any purpose in animals can be associated with the increase of antibiotic resistance that can also be promoted by the co-selection by different types of antimicrobials, a relevant aspect in multidrug resistant bacteria.

All isolates carried the *tet*A gene, except one with the *tet*B gene. The *tet*A gene has been detected in *E. coli* isolates obtained in Algeria from the food liver samples in the same region of this study (Djelfa) [27], and from retail raw ground beef [59]. In a study carried out previously by our group on broilers in the Bejaia city, the use of tetracycline was found to be frequent and abundant, which was probably behind the high rates of resistance towards this antibiotic [60]. Antibiotic resistance detected in *E. coli* isolates of sparrow might suggests potential transfer of antibiotic resistant bacteria from farm animals to sparrows. This migratory bird can be a reservoir and a vector in the transference of antimicrobial resistant bacteria.

The *sul1* gene was found in *E. coli* isolates from turkey and chicken. It was also reported from *E. coli* in food liver samples in Djelfa city, Algeria [27].

Integrons, which constitute a gene capture system capable of effectively promoting the dissemination of antibiotic resistance genes within the bacterial world, were found in 10 *E. coli* isolates from sparrow and turkey, defined by the presence of *intl1* gene. It was also detected by Chenouf et al., (2021) from food liver samples [27]. The role of these genetic elements in the dissemination of resistant bacteria is known, given that most gene cassettes encode antibiotic resistant determinants.

## 5. Conclusions

Important ESBL encoding genes (blaCTX-M-14, blaCTX-M-15 and blaCTX-M-1) were detected in E. coli isolates from broiler, turkey, and sparrow samples in Algeria, with the diversity of genetic lineages of the isolates. These results provide evidence that antibiotic resistance is not limited to humans and domesticated animals. Wild animals (sparrow in our case) could have a role in the dissemination of antibiotic resistance genes.

## Figures and Tables

**Table 1:** Phenotypic and genotypic characteristics of the 12 ESBL-producing *E. coli* isolates recovered of sparrow, turkey and broiler faecal samples.

Isolate Code	Origin	Animal Specie	Phylogenetic Group/ST (CC)	Antimicrobial Resistance Phenotype	ESBL Enzyme	Other Resistance Genes
X1998	Mergueb (Djelfa)	Sparrow	B1/ST602	CTX, NAL, CIP, TET	CTX-M-14	*tetA, intl1*
X1999	Mergueb (Djelfa)	Sparrow	B1	CTX, NAL, CIP, TET	CTX-M-14	*tetA, intl1*
X2000	Mergueb (Djelfa)	Sparrow	B1	CTX, NAL, CIP, TET	CTX-M-14	*tetA, intl1*
X2001	Mergueb (Djelfa)	Sparrow	B1	CTX, NAL, CIP, TET	CTX-M-14	*tetA, intl1*
X2002	Mergueb (Djelfa)	Sparrow	B1	CTX, ATM, NAL, CIP, TET	CTX-M-14	*tetA, intl1*
X2004	Mergueb (Djelfa)	Sparrow	B1	AMC, CTX, CAZ, ATM, NAL, CIP, TOB, TET	CTX-M-14	*tetA, intl1*
X2005	Mergueb (Djelfa)	Sparrow	B1	CTX, ATM, NAL, CIP, TOB, TET	CTX-M-14	*tetA, intl1*
X2006	Mergueb (Djelfa)	Sparrow	B1	CTX, ATM, NAL, CIP, TOB, TET	CTX-M-14	*tetA, intl1*
X2013	Rous El Ayoun (Djelfa)	Turkey	D	AMC, CTX, TET	CTX-M-15	*tetA*
X2016	Zaafrane (Djelfa)	Turkey	D/ST4492 (CC405)	AMC, CTX, CAZ, ATM, NAL, CIP, TOB, SXT, TET	CTX-M-15	*tetB, intl1*
X2018	Zaafrane (Djelfa)	Turkey	D	CTX, ATM, NAL, CIP, SXT, TET	CTX-M-14	*tetA, sul2, intl1*
X2023	Driaat (M’Sila)	Chicken	D/ST117	CTX, NAL, CIP, TET	CTX-M-1	*tetA, sul2*

AMC: amoxicillin-clavulanic acid; CTX: cefotaxime; CAZ: ceftazidime; ATM: azthreonam; NAL: nalidixic acid; CIP: ciprofloxacin; TOB: tobramycin; SXT: trimethoprim-sulfamethoxazole; TET: tetracycline.
